# In Vitro Systems for the Study of Hepatitis C Virus Infection

**DOI:** 10.1155/2012/292591

**Published:** 2012-09-27

**Authors:** Garrick K. Wilson, Zania Stamataki

**Affiliations:** NIHR Biomedical Research Unit and Centre for Liver Research, School of Immunity and Infection, College of Medical and Dental Sciences, University of Birmingham, Birmingham B15 2TT, UK

## Abstract

The study of a virus is made possible by the availability of culture systems in which the viral lifecycle can be realized. Such systems support robust virus entry, replication, assembly, and secretion of nascent virions. Furthermore, culture models provide a platform in which therapeutic interventions can be devised or monitored. Hepatitis C virus (HCV) has a restricted tropism to human and chimpanzees; thus investigations of HCV biology have been hindered for many years due to a lack of small animal models. Nevertheless, significant efforts have been directed at developing cell culture models to elucidate the viral lifecycle in vitro. HCV primarily infects liver parenchymal cells commonly known as hepatocytes. The liver is a highly specialized and complex organ and the development of in vitro systems that reflects this complexity has proven difficult. Consequently, host cell receptor molecules that potentiate HCV infection were identified over a decade after the virus was discovered. A summary of the various HCV in vitro culture models, their advantages, and disadvantages are described.

## 1. Introduction

HCV infection is a major concern for human health with an estimated 3% of the world's population infected [1]. HCV primarily infects hepatocytes in the liver culminating in serious and progressive liver disease including chronic hepatitis, cirrhosis, and hepatocellular carcinoma. The established treatment for HCV infection is a combination of interferon-*α* and ribavirin. However, only a fraction of patients respond to this treatment; moreover side effects including fatigue and depression are commonplace. More recently, two NS3 protease inhibitors Boceprevir and Telaprevir have been licensed for HCV treatment [2, 3]; however, there are reports of virus resistance to both inhibitors [4]. In cases of HCV-associated liver failure, transplant is the only therapy, making HCV a leading indicator for liver transplantation in the developed world. However, circulating virus in the blood results in reinfection of the newly transplanted liver in all cases consistent with an aggressive course of accelerated hepatic histological changes [5]. HCV-related graft complications are associated with a significant reduction in patient survival and is the leading cause of liver failure in many transplant centres [6]. 

## 2. HCV Biology

HCV resembles the Flaviviridae family of viruses and is the sole member of the *Hepacivirus* genus. Virus particles range between 50 and 60 nm in diameter; each virion is comprised of a lipid bilayer envelope bearing the E1E2 glycoprotein complex that facilitates particle and host cell interactions. The envelope surrounds a capsid that contains a positive sense single-stranded RNA genome approximately 9,600 nucleotides long encoding a 3000-amino-acid polyprotein. The polyprotein is posttranslationally modified by host and viral proteases into three structural proteins (Core, E1, and E2), which are the capsid and envelope glycoproteins, respectively, a small ion channel (p7) and six nonstructural proteins (NS2, NS3, NS4A, NS4B, NS5A, and NS5B). The structural proteins comprise the building blocks for the virion and the nonstructural proteins replicate the viral RNA [7]. Following an initial association with a series of attachment factors, including C-type lectins, lipoprotein receptors, and heparan sulfate (reviewed in [8]), HCV infects hepatocytes via four cellular receptor molecules. These are the tetraspanin CD81, scavenger receptor class B member I (SR-BI), and the tight junction proteins Claudin-1 and Occludin [9–12]. HCV enters the cell via clathrin-mediated endocytosis followed closely via viral envelope uncoating and release of the RNA genome into the cytoplasm where translation and replication occur on specialized endoplasmic reticulum (ER) membrane webs. Virion assembly and secretion are associated with the lipoprotein pathway accounting for the lipid-rich nature of serum-derived HCV particle; the HCV lifecycle is reviewed in [8]. 

## 3. Model Systems to Study HCV Biology

### 3.1. HCV Replicons

To study HCV replication, Lohmann and colleagues created mini-HCV genomes called replicons (genetic elements that can replicate autonomously) from the liver RNA of a chronically infected patient [13]. HCV replicon constructs were able to replicate autonomously when introduced into several hepatoma cell lines allowing for the identification of permissive cell types and adaptive mutations that promote HCV replication in vitro [14–16]. The replicon system made it possible to study host and viral signaling necessary for virus replication and echoed a new dawn in HCV research. However, the process of viral entry and assembly could not be studied using this in vitro model.

### 3.2. HCV Pseudoparticles

One approach employed by researchers to realize how HCV interacts with host cells was to express the viral encoded glycoproteins (E1E2) in isolation from other viral encoded proteins. However, high-level expression of E1E2 resulted in misfolded aggregates [17]. To overcome this, researchers expressed chimeric glycoproteins incorporating transmembrane regions of E1E2 known to be expressed at the plasma membrane or truncated glycoproteins lacking transmembrane domains [18]. Deletion of the HCV E2 transmembrane domain resulted in the secretion of a soluble form of E2 (sE2) [19]; sE2 was used to identify two putative HCV receptors (SR-BI and CD81). The identification of two putative receptors using sE2 indicated that E2 is the major glycoprotein responsible for receptor binding. However, E1E2 exists as heterodimers suggesting that sE2 was unlikely to recapitulate functional HCV glycoproteins. 

The development of infectious HCV pseudoparticles (HCVpp) enabled studies of the entry aspect of the virus lifecycle [20]. Pseudoparticles take advantage of the ability of retroviruses to incorporate heterologous glycoproteins in their membrane during budding. HCVpp infection of hepatoma cells was ablated by specific E1 and E2 neutralizing reagents to confirm that both E1 and E2 are indispensable for HCV entry [20–22] and provided the first functional assay to screen the effects of neutralizing antibodies on virus entry. HCVpp can also infect primary hepatocytes with infectivity measureable utilizing various reporter systems, yet the levels of infection are usually lower than hepatocarcinoma cell lines and are subject to interdonor variation [22]. The HCVpp system was critical in the identification of HCV coreceptors Claudin-1 and Occludin and to date continues to aid in our understanding of HCV binding, attachment, and internalization. 

### 3.3. Cell-Culture-Derived HCV (HCVcc)

2005 hallmarked a major breakthrough in HCV research. Several laboratories reported an HCV strain that replicates and releases infectious particles in cell culture (HCVcc) [23–25]. The strain was cloned from a genotype 2a virus isolated from a Japanese patient with severe acute HCV infection. This unique clone was referred to as Japanese Fulminant Hepatitis 1 (JFH-1). HCVcc meant that the full viral lifecycle could be studied in vitro. Unlike all previous HCV genomes tested, JFH-1 infection of hepatoma cells resulted in the release of progeny virus capable of infecting naive cells. Importantly, HCVcc was infectious for chimpanzees and mice transplanted with human hepatocytes [26]. HCVcc confirmed major findings made using HCVpp including the identification of HCV co-receptors and continues to increase our understanding of the HCV lifecycle. The successful isolation of JFH-1 paved the way for the development of several chimeric HCVcc constructs representing diverse genotypes [27–29]. Clinical manifestations of hepatitis, such as cirrhosis and hepatocellular carcinoma, are typically associated with genotype 1 HCV, which is more prevalent than genotype 2 and relatively resistant to IFN therapy. A fully replicating HCVcc system from a genotype 1a virus has been developed (H77-S) and was also infectious for chimpanzees, yet this isolate was not as infectious as JFH-1 in vitro [30]. In conclusion, our understanding of HCV has been hindered for many years primarily because of a lack of robust model systems to study the virus lifecycle. The development of in vitro systems has greatly enhanced our understanding of key aspects in the virus lifecycle.

### 3.4. In Vitro Cell Culture Systems

Since the identification of the viral genome 23 years ago [31], significant progress has been made in delineating model systems to study the viral lifecycle in vitro. However, our understanding of HCV pathogenesis is still in its infancy; to date, the search continues for physiologically relevant cell culture models to study authentic host response to virus infection. This has been compounded by difficulties in developing cell lines that recapitulate the intricacy of the liver microenvironment. 

### 3.5. Primary Cell Culture and Immortalized Primary Hepatocytes

Attempts to propagate HCV in vitro have proven difficult; initial studies capitalized on the hypothesis that virus infection was dependent on host factors expressed in highly differentiated hepatic cells. Hepatocytes in vivo are quiescent and primary hepatocytes in culture demonstrate minimal cell division. As such, primary hepatocyte cultures from humans or chimpanzees chronically infected with HCV were utilized for HCV studies. However, the use of primary cell cultures were inadequate for several reasons; they supported low levels of HCV replication, heterogeneous virus populations and HCV-specific antibodies in the sera of infected patients impaired the levels of infection, and the system suffered from poor data reproducibility (reviewed in [32, 33]). In many cases HCV replication was assessed by reverse transcriptase polymerase chain reaction (RT-PCR) to detect HCV RNA levels which was indicative of virus replication [34]. This technique proved useful in detecting low levels of HCV RNA; however, it also presented new challenges including the potential for random priming by cellular nucleic acids, contamination of RNA samples, and lack of strand specificity due to RNA self-priming [35, 36]. As such additional criteria were introduced to validate HCV replication, these included treatment of infected cells with interferon-*α* (IFN*α*) to cure from viral RNA and sequence analysis to demonstrate genome variability.

More recently key aspects of the HCV lifecycle have been delineated in primary hepatocytes, including pH-dependent virus entry using the HCV pseudoparticle system (HCVpp) and clathrin-mediated endocytosis [37, 38]. A study by Podevin et al. reported the production of significant titres of infectious HCV particles in adult primary hepatocytes. Importantly, secreted particles demonstrate a low buoyant density and high specific infectivity, which is similar to HCV particles produced in vivo. These findings suggest that primary hepatocytes represent a physiological in vitro model to study HCV infection [38]. Despite the successes with primary hepatocyte cultures their lifespan in culture is relatively short, concomitant with a decrease in hepatocyte differentiation phenotype. 

Researchers have employed a number of techniques to maintain liver-specific functions in isolated hepatic cells in vitro [39]. These parameters include 3D cell culture to increase cell-cell interactions, extracellular matrix deposition, and coculture of different cell types [39–41]. To study HCV infection of normal hepatocyte biology Ploss and colleagues utilized a microscale model of the human liver that maintained hepatic phenotypic for several weeks in culture. The system is comprised of primary hepatocytes organized into colonies surrounded by stromal cells [42]. The so-called micropatterned co-culture model supported the entire HCV lifecycle with sustained viral RNA replication for several weeks. Importantly, the model formed polarized cell layers, which is a key feature of hepatocyte physiology. This system may therefore provide a platform for the assessment of anti-HCV therapeutics; nevertheless, micropatterned co-culture is not widely available and primary hepatocytes in general are difficult to obtain due to shortages in donor liver specimens. Furthermore, inherent donor liver variations may affect the HCV lifecycle making it difficult to reproduce findings with primary cells. 

Several reports have utilized human pluripotent stem-cell-derived hepatocytes to study various aspects of the HCV lifecycle. Pluripotent stem cells have the ability to produce an unlimited source of nontransformed differentiated cells. Indeed, hepatocytes from this system demonstrate liver metabolic activity and hepatocyte phenotypic markers and are an attractive alternative for primary hepatocytes. Stem-cell-derived hepatocytes express all four HCV receptor molecules and support persistent HCV infection including the complete replication cycle and the release of progeny infectious virus. In addition, HCV replication was blocked using specific inhibitors [43–46]. Importantly, HCV infection of stem-cell-derived hepatocytes induced an antiviral inflammatory response including the production of interferon genes [47]. 

It is believed that stem cell hepatocytes offer a physiologically relevant system to study HCV biology. Nevertheless, despite the demonstration of liver metabolic activity, it is important to point out that they are not hepatocytes per se; they are perhaps best described as hepatocyte-like since they were induced from either an embryonic lineage or via reprogramming with various factors. Even so, they represent an important breakthrough in HCV studies offering new opportunities for the identification of signaling pathways required for virus infection. Furthermore, apart from primary hepatocytes they are arguably the best alternative approach for an in vitro model that mimics hepatocytes in vivo. 

Lázaro et al. reported HCV replication in nontransformed human fetal hepatocytes, which maintained and secreted HCV particles for 2 months after transfection [48]. Similarly, two recent reports have shown HCV infection of primary human fetal liver cells (HFLCs) [47, 49]. The authors reported the induction of interferon-stimulated genes (ISGs) in response to HCV infection and concluded that this model provides a useful surrogate to study HCV gene induction in vivo. However, there were significant variations in ISG expression and HCV infection of the different donor fetal cells. 

Due to the aforementioned difficulties using primary cell types, scientists have developed immortalized hepatocyte cell lines. PH5CH and HuS-E cells were generated by immortalizing primary hepatocytes with the T antigen of simian virus 40 and the E6/E7 genes of the human papillomavirus, respectively [50, 51]. Although these cells supported HCV replication, the production of virus particles was restricted and the levels of RNA replication were low, making them nonviable for long-term studies. 

## 4. Hepatoma Cell Lines

### 4.1. Huh-7 Clones

The HCV replicon system made it possible to identify permissive hepatoma cell lines that support efficient HCV replication. Blight and colleagues transfected Huh-7 hepatoma cells with subgenomic replicons and selected cells containing replicating RNA for prolonged interferon-*α* treatment to cure cells of the viral RNA. Sustained interferon-*α* treatment resulted in clonal populations that were tested for their ability to support HCV replication after retransfection with HCV replicons [14]. One cell clone in particular, denoted Huh-7.5, showed a significant enhancement in HCV replication compared to other clones. Efficient virus replication in Huh-7.5 cells was partly attributed to a defective retinoic-acid-inducible gene-I (*RIG-I*) pathway, which is essential for an antiviral immune response [52, 53]. The discovery of Huh-7 clones (Huh-7, Huh-7.5, and Huh-7.5.1) echoed a new dawn in HCV research. To date, much of our understanding of HCV biology is shaped through the use of these cells. The HCVpp and HCVcc systems are capable of infecting Huh-7 cells at significantly high levels and key events including virus entry, replication, and secretion have been deduced. These cells continue to be widely used as they are of hepatic origin, highly susceptible to virus infection, and are arguably the best available in vitro host for HCV to date. However, Huh-7 clones are derived from a human hepatocellular carcinoma. As such they are poorly differentiated, display abnormal proliferation, aberrant gene regulation, and altered signaling pathways raising questions about their physiological relevance to the in vivo environment [54]. 

Attempts have been made to improve the differentiation status of Huh-7 cells. As discussed, hepatocytes in vivo are nondividing and Huh-7 cells in culture demonstrate asynchronous cell division. To impair cell division and improve the differentiation status of Huh-7 cells, Sainz and Chisari treated Huh-7 cells with Dimethyl Sulfoxide (DMSO), which has been reported to inhibit cell growth and improve hepatocyte differentiation. DMSO treatment resulted in cytogenetically differentiated Huh-7 cells that were non-dividing, characterized by increased expression of hepatocyte differentiation markers. Moreover, cells were capable of supporting persistent HCV infection [55]. Differentiated cells supported infection in the presence of type I and type III interferon antiviral treatment, resembling persistence in patients [56]. DMSO-treated hepatoma cells may therefore more accurately mimic HCV infection of the in vivo environment. 

### 4.2. Other Hepatomas

Several other hepatoma cell lines have been reported for HCV infection. Hep3B cells are derived from a hepatocellular carcinoma and express all four major HCV receptor proteins. They support high levels of HCV entry concomitant with reduced viral RNA replication [57]. Huh-6 cells are derived from a hepatoblastoma and express low levels of Claudin-1 making them nonpermissive for HCV infection. However, ectopic expression of Claudin-1 induced susceptibility to virus entry with limited replication suggesting that intrinsic cellular factors may be anti-viral at a postentry level in these cells [58]. Interestingly, one study has shown that naïve Huh-6 cells support efficient viral RNA replication when transfected with HCV replicons. Furthermore, Huh-6 cells are highly resistant to interferon-*γ* treatment making them a potential tool to study anti-viral compounds in vitro [59]. Other studies have utilized the PLC/PRF/5 cell line. PLC/PRF/5 cells were obtained from a primary liver carcinoma and support HCVpp entry that was 3 times higher compared to Huh-7 cells [60, 61]. These cells proved useful in dissecting HCV internalization via clathrin-mediated endocytosis. Unfortunately, PLC/PRF/5 cells were not permissive for HCVcc infection in the same study [60]. More recently, Sainz and colleagues assembled a panel of hepatic cell lines to compare their ability to support HCV infection [57]. The authors reported comparable HCVpp entry into Hep3B, PLC/PRF/5, and Huh-7 cells. However, later steps in the viral lifecycle including replication were impaired to different degrees in Hep3B and PLC/PRF/5 cultures compared to Huh-7 cells. Notably, there was a significant increase in ISG56 expression in Hep3B and PLC/PRF/5 cells in response to virus infection. 

Taken together, these studies suggest that the majority of hepatoma cell lines support HCVpp entry. However, later steps in the viral lifecycle are inefficient. An innate immune response may reduce HCV activity at a post entry level to varying degrees in the different cell types. Furthermore, it is possible that cellular factors including micoRNA-122 which is important for HCV replication [62] demonstrate reduced endogenous expression in most hepatoma cell lines compared to highly permissive Huh-7 clones. Our understanding of the HCV lifecycle would benefit from the identification of additional permissive cells and further studies to address the endogenous expression of cellular factors important for the HCV lifecycle may prove enlightening. A list of commonly used in vitro models to study the biology of HCV is listed in Table 1. 

### 4.3. Polarization 

The discovery of Claudin-1 and Occludin as HCV entry factors highlighted the importance of studying hepatocyte polarity in HCV infection. Hepatocytes in vivo demonstrate a complex polarity with tight junction proteins separating the apical canalicular domain from the basolateral sinusoidal membrane. Each membrane is associated with a specific protein and lipid profile that is crucial to the correct functioning of the liver. Unfortunately, there are limited polarized cell types of hepatic origin that support efficient HCV infection. As such, there is a real need for cells that demonstrate hepatocyte-like polarity and support high levels of HCV infection. Cultured primary hepatocytes demonstrate a simple epithelial polarity similar to the phenotype seen in most polarized epithelial cell lines including Caco2 and MDCK (unpublished observations). Moreover, they rapidly lose this phenotype accompanied by a loss of tight and adherens junctions [71]. Micropatterned co-cultures of primary hepatocytes developed polarized membranes and the localization of HCV entry factors was similar in human liver tissue [42]. In our experience Huh-7 cells fail to polarize in culture suggesting that they are unlikely to reflect polarized hepatocytes in vivo [69]. This is in contrast to a report by Yang et al. showing that Huh-7 cells display transepithelial resistance, consistent with a polarized phenotype [72]. The differences may simply reflect variations between laboratory clones of Huh-7 cells, as previously shown [73]. Recently, a 3D matrigel-embedded Huh-7 cell culture system has been described [74]. In this system Huh-7 cells developed so-called proto-bile canaliculi structures indicative of hepatocyte polarization and supported HCV infection. 

Mee et al. utilized the polarized Caco2 cells, which express all four HCV receptors and supported HCV infection, to study the effects of polarity on virus infection. Tight junctions create a barrier that restrict HCV entry into Caco2 cells and disruption of these junctions increased virus infection suggesting that polarity may reduce HCV infection of hepatocytes [69]. Indeed, a follow-up study by the same authors studied the effect of polarity on HCV entry in the polarized hepatoma cell line HepG2 [75]. HepG2 cells are derived from a human hepatoblastoma and express liver-specific metabolic proteins such as the canaliculi marker MRP2 (multi-drug-resistant protein −2) and Bsep (bile salt export protein) [71]. They form polarized cell membranes over time in culture consistent with the development of apical lumens that constitute the apical bile canaliculi [71]. Naïve HepG2 cells do not express CD81; however, complementation with exogenous CD81 (HepG2-CD81) induces susceptibility to HCV infection, although the level of infection in these cells is 724-fold reduced compared to Huh-7.5 cells. Nevertheless, HepG2-CD81; cells allowed detailed study on the effects of polarization of virus entry, whereby there is an inverse correlation between virus entry and increasing polarity [75]. Importantly, the pattern of HCV receptor distribution in these cells is similar to observations made in human liver specimens.

One of the greatest challenges since the discovery of HCV is to ascertain the effects of HCV infection on hepatocyte biology. As such the mechanism(s) underlying HCV perturbation of hepatocyte physiology are largely unexplained. Studies utilizing HepG2 cells have provided insights into the functional consequences of virus infection on hepatocellular biology. HCV infection of HepG2-CD81 cells induced a loss of polarity and tight junction integrity in a vascular-endothelial-growth-factor-(VEGF-) dependent manner [65]. We have recently reported that HCV infection of HepG2-CD81 cells induces a cellular dedifferentiation state reminiscent of epithelial to mesenchymal transition (EMT) via a hypoxia-inducible-factor-1*α*-(HIF-1*α*-) dependent perturbation of cellular homeostasis [76]. The mechanism(s) by which HCV promotes liver injury are unclear, as the virus does not integrate with the host DNA. VEGF, EMT, and HIF-1*α* signaling are intrinsically liked with tumorigenesis including hepatocellular carcinoma. Therefore, the use of HepG2 cells has aided in our knowledge of the pathways underlying HCV-induced liver injury. 

### 4.4. Liver Slices

In the pursuit of experimental models that represent physiological and pathological conditions that support HCV infection. Lagaye et al. reported an ex vivo model based on human adult liver slices for HCVinfection [77]. The authors demonstrated for the first time the ability of liver tissue to support de novo virus replication and the production of infectious HCV particles. Furthermore, viral infection was neutralized with anti-CD81 or anti-E2 antibodies in a dose-dependent manner to demonstrate an HCV-specific effect [77]. The system provides a close match to the hepatic microenvironment and may prove useful to study virus spread in the liver parenchyma. Ex vivo liver slices have been previously used to validate in vitro experiments in a noninfectious context [78]. Disadvantages of the use of liver slices for HCV research include restricted availability and short-term viability of the samples. Nevertheless, this ex vivo model is the closest we have approached to mimicking authentic liver function for the study of HCV and can be amenable to the study of antivirals [77].

## 5. Conclusions

The restricted tropism of HCV and lack of small animal models have necessitated the development of in vitro model systems for the study of the full virus cycle. Since the virus was first cloned over 20 years ago [31], in vitro replicating clones have become available and with these coevolved novel permissive in vitro platforms to enable investigation of infection, transmission, and therapeutic interventions. The routine culture of patient-derived viruses remains elusive and novel approaches are required to achieve this ultimate goal. We recently demonstrated that nonpermissive B lymphocytes can act as vehicles for HCVcc transmission to hepatoma cell lines, delivering.

 infection with higher specific infectivity than cell-free virus [79]. This mode of transmission, utilizing vector cells as “Trojan horses” for the infection of target cells, has been described previously for other viruses [80, 81]. Despite the lack of evidence for in vivo significance, these models are advantageous for in vitro infection. Further advances in the development of in vitro and ex vivo models for the study of HCV infection in a manner that closely mimics the liver are greatly anticipated, especially to evaluate the efficacy of promising new direct acting antiviral treatments and host-targeted agents currently in the pipe line [82].

## Figures and Tables

**Table 1:** Culture models to study the HCV lifecycle. Several hepatic models have been employed to study diverse aspects of the viral lifecycle. Models of nonhepatic origin have also been employed and proved useful in identifying potential extra hepatic sites of HCV infection in vivo. In addition, Caco2 cells were used to study the effects of polarity on HCV infection as most hepatic cell lines fail to polarize in culture. However, a perfect model that closely mimics the in vivo environment is still yet to be identified.

Cell type	Tissue	HCV lifecycle	Reference	Comment
Immortalized cell lines				
Huh 6/Claudin-1	Hepatoblastoma	Entry/replication	[58, 59]	Interferon resistance
Huh-7	HCC	Full lifecycle	[14]	Interferon response to infection
Huh-7.5	HCC	Full lifecycle	[14]	Defective RIG-I pathway
Hep3B	HCC	Entry	[57, 63]	Limited HCV replication
HepG2-CD81	Hepatoblastoma	Entry/replication	[64–66]	Forms hepatic polarity
PLC/PRF/5	Primary liver carcinoma	Entry	[57, 60]	Low HCV replication
293-T/Claudin-1	Kidney	Entry/replication	[11]	Non-hepatic origin
hCMEC/D3	Brain endothelia	Entry/replication	[67]	In vitro study of HCV neuropathology
HBMEC	Brain endothelia	Entry/replication	[67]	In vitro study of HCV neuropathology
SK-N-MC	Neuroepithelioma	Entry	[68]	Low virus replication
SK-PN-DW	Neuroepithelioma	Entry	[68]	Low virus replication
Caco2	Colorectal adenocarcinoma	Entry/replication	[69]	Non-hepatic origin Demonstrates epithelial polarity

Primary cell culture				

Primary hepatocytes	Human liver	Full lifecycle	[38, 42, 70]	Limited access to liver tissue, physiologically relevant model
Fetal hepatocytes	Human liver	Entry/replication	[47–49]	Limited access to liver tissue, antiviral response to infection
Chimpanzee hepatocytes	Chimp liver	Replication	[36]	Limited availability
Stem cell derived hepatocytes	Human embryo	Full life cycle	[43, 44, 46]	Highly differentiated, supports HCV life cycle
